# Dynamic Meta-data Network Sparse PCA for Cancer Subtype Biomarker Screening

**DOI:** 10.3389/fgene.2022.869906

**Published:** 2022-05-09

**Authors:** Rui Miao, Xin Dong, Xiao-Ying Liu, Sio-Long Lo, Xin-Yue Mei, Qi Dang, Jie Cai, Shao Li, Kuo Yang, Sheng-Li Xie, Yong Liang

**Affiliations:** ^1^ Institute of Systems Engineering, Macau University of Science and Technology, Avenida Wai Long, Taipa, China; ^2^ Computer Engineering Technical College, Guangdong Polytechnic of Science and Technology, Zhuhai, China; ^3^ MOE Key Laboratory of Bioinformatics, TCM-X Center/Bioinformatics Division, BNRIST/Department of Automation, Tsinghua University, Beijing, China; ^4^ Guangdong-HongKong-Macao Joint Laboratory for Smart Discrete Manufacturing, Guangzhou, China; ^5^ Peng Cheng Laboratory, Shenzhen, China

**Keywords:** Cancer subtype, biomarkers, sparse PCA, DM-ESPCA model, meta-data, dynamic network

## Abstract

Previous research shows that each type of cancer can be divided into multiple subtypes, which is one of the key reasons that make cancer difficult to cure. Under these circumstances, finding a new target gene of cancer subtypes has great significance on developing new anti-cancer drugs and personalized treatment. Due to the fact that gene expression data sets of cancer are usually high-dimensional and with high noise and have multiple potential subtypes’ information, many sparse principal component analysis (sparse PCA) methods have been used to identify cancer subtype biomarkers and subtype clusters. However, the existing sparse PCA methods have not used the known cancer subtype information as prior knowledge, and their results are greatly affected by the quality of the samples. Therefore, we propose the Dynamic Metadata Edge-group Sparse PCA (DM-ESPCA) model, which combines the idea of meta-learning to solve the problem of sample quality and uses the known cancer subtype information as prior knowledge to capture some gene modules with better biological interpretations. The experiment results on the three biological data sets showed that the DM-ESPCA model can find potential target gene probes with richer biological information to the cancer subtypes. Moreover, the results of clustering and machine learning classification models based on the target genes screened by the DM-ESPCA model can be improved by up to 22–23% of accuracies compared with the existing sparse PCA methods. We also proved that the result of the DM-ESPCA model is better than those of the four classic supervised machine learning models in the task of classification of cancer subtypes.

## Introduction

As the most difficult-to-cure malignant disease in the world, how to defeat cancer has received extensive attention from researchers (Siegel et al., 2016; Siegel et al., 2019). The latest research shows that each type of cancer can derive many subtypes, which may be one of the reasons why personalized cancer treatment is needed (Nguyen et al., 2008; Cancello et al., 2010; Houssami et al., 2012; Symmans et al., 2017; and Waks and Winer, 2019). For example, the ceritinib capsule is a targeted drug for lung cancer (the target gene is ALK) (Cooper et al., 2015; Raedler, 2015). However, existing studies have shown that it only has a good effect on a small number of lung cancer patients. The reason for this problem is that only 35–36% of lung cancer patients are caused by ALK gene mutations, which means that the ceritinib capsule is only effective for one subtype of lung cancer (Deeks, 2016). Therefore, the identification and recognition of potential target genes corresponding to cancer subtypes have become an important task in cancer research (Banerji et al., 2012; Calon et al., 2015; and De Cecco et al., 2015).

With the rapid development of the high-throughput sequencing technology, there are a lot of biological data that have been collected from many large-scale projects, which provides a basis to establish machine learning models for biomarker screening. At present, there are two types of machine learning models for screening target genes of potential cancer subtypes. One is the supervised classification models. Gene expression data sets of cancer are usually high-dimensional and with high noise and small sample sizes, which easily lead to overfitting of supervised machine learning models (Gao et al., 2019; Lee et al., 2020). Moreover, the other problem with the supervised models is that the gene probes screened by these models may not have good biological interpretation, and different models may screen out very different gene probes in the same data set (Xie et al., 2019; Yang et al., 2019). The other type is the unsupervised biomarker extraction models. The principle of these models is to perform cancer subtype clustering and target gene screening based on potential patterns of samples. Among them, the sparse principal component analysis (sparse PCA) methods are widely used methods of unsupervised biomarker extraction, which can capture the linear relationship of variables to best explain the latent patterns of cancer subtypes. Moreover, the potential target genes screened by the sparse PCA methods may tend to have good biological interpretability (Shen et al., 2009; Shen et al., 2012; and Min et al., 2018).

Currently, researchers have proposed some sparse PCA and joint latent variable methods for identifying driver genes of cancer or biomarkers of cancer subtypes. For example, in 2009, Shen et al. (2009) proposed a cancer subtype clustering model (iCluster) based on joint latent variable of data. In 2011, SAN et al. (Navarro Silvera et al., 2011) used PCA and logistic regression to analyze the risk factors of esophageal cancer and gastric cancer. Shen et al. (2013) further extended the iCluster model with LASSO, elastic net, and fusion LASSO methods to allow feature selection in an integrated clustering environment. The overall goal of these models is to obtain joint clustering of samples and identify cluster-related features across data sets. In 2015, Sill et al. (2015) proposed a sparse PCA method (S4VDPCA) with stable selection ability to process the medulloblastoma brain gene expression data set and revealed that the genes determined by the first two sparse PC loadings significantly participated in the marrow and several key pathways between the molecular subgroups of blastoma. In 2018, Min et al. (2018) proposed an edge group sparse PCA model (ESPCA) which effectively enhanced the potential gene selection ability of sparse PCA. Existing research shows that structured sparse models similar to ESPCA can effectively improve the biological interpretability and feature selection capabilities of the models (Min et al., 2016; Min et al., 2019; Vinga, 2021).

However, the existing sparse PCA methods still have three main issues. First, all these methods are reference-free methods, which means that they do not consider the known subtype classification information of the cancer data set (Reis-Filho and Pusztai, 2011; Dai et al., 2015). The existing research works have shown that reference-free sparse PCA methods may discard some potential biomarkers in the process of sparseness (Kim et al., 2019). The second one is that the samples of the biological data contain a lot of noise (Teng, 2003; Linck and Battey, 2019), which will affect the final results of the model and eventually lead researchers to find the wrong potential target gene. The third issue is that most of the existing sparse PCA methods use the greedy optimization principle to select target gene probes, which will make the model fall quickly into a local optimum.

**FIGURE 1:**
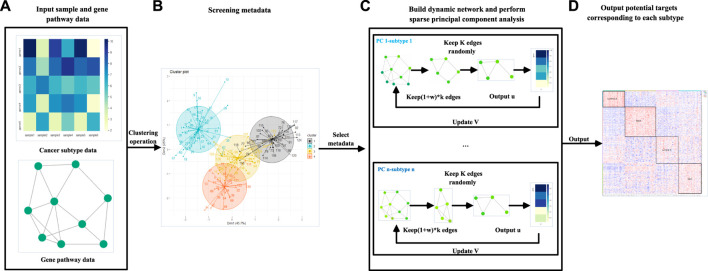
Flow chart of the DM-ESPCA model. **(A)** The DM-ESPCA model requires input gene expression and pathway data. **(B)** The DM-ESPCA model selects meta-data by clustering all samples. **(C)** Workflow of the DM-ESPCA model to screen targeted genes. The DM-ESPCA model will generate a dynamic gene network for each subtype. **(D)** Finally, this model will output the screened genes.

In order to solve the three problems mentioned mentioned above, this article proposes the DM-ESPCA model, which uses the dynamic gene network, meta-learning approach, and random sampling algorithm based on the greedy principle (Figure 1). The purpose of the dynamic gene network is to enhance the feature selection ability of the model to screen out potential target genes that are more relevant to the cancer subtype. The meta-learning approach is an efficient machine learning framework, which uses a small number of high-quality samples to adjust the parameters of the machine learning model to reduce the errors caused by the noise data. We also proposed a random sampling algorithm based on the greedy principle to obtain a better solution in the process of sparseness.

The steps of the DM-ESPCA model are as follows: 1) filter meta-data for each subtype in the cancer data set; 2) based on meta-data, use known subtype classification information as prior knowledge to calculate the correlation degree of each gene probe corresponding to each subtype; 3) use the quantitative value of correlation as a parameter to generate a unique biological network for each subtype; and 4) build the DM-ESPCA model using the dynamic gene network to screen biomarkers for each subtype.

This article conducted experiments on three data sets, and the results showed that the DM-ESPCA model is better than the existing sparse PCA methods. The heat maps and bio-enrichment analyses show that the potential target genes screened by the DM-ESPCA model have higher correlations and richer biological information with the corresponding cancer subtypes. The results of re-clustering and the accuracies of machine learning classification models based on the potential target genes screened by the DM-ESPCA model can be improved by up to 23 and 22%, respectively.

## Materials and Methods

### Data Sets

In this experiment, we used three cancer data sets to test the performance of the DM-ESPCA model, including two breast cancer data sets and one gastric cancer data set. All these data sets were assayed with the Human Genome U133 Plus 2.0 microarray (HG-U133_Plus_2). This gene chip contains 54,675 probes (Carlson et al., 2016). The following is a detailed introduction to the data sets (Table 1):

**TABLE 1:** Details of the three data sets.

	BCI	BCII	GC
Number of samples	155	178	70
Number of genes	54,675	54,675	54,675
Number of subtypes	4	4	5
ID	E-GEOD-45827	E-GEOD-65194	E-GEOD-35809

First, we used a breast cancer subtype data set, numbered E-GEOD-45827 (BCI, https://www.ebi.ac.uk/arrayexpress/experiments/E-GEOD-45827/). Since breast cancer is a kind of malignant cancer, its incidence rate ranks first among female malignant cancers all year round and is still increasing year by year (DeSantis et al., 2014; Fan et al., 2014). Therefore, the analysis of breast cancer data sets is greatly significant. Meanwhile, breast cancer has a clear subtype division, which is mainly divided into four subtypes, including Basal, Her2, Luminal A, and Luminal B (Tran and Bedard, 2011). The BCI data set we used in this experiment contains 155 samples (Supplementary Fig.1.A).

Next, we used another breast cancer data set, numbered E-GEOD-65194 (BCII, https://www.ebi.ac.uk/arrayexpress/experiments/E-GEOD-65194/). The purpose of using the BCII data set is to verify whether our proposed model can correctly classify the subtypes and whether it has sufficient stability in the same cancer but different batches of data collection. Here, the BCII data set also has four subtypes, including TNBC, Her2, Luminal A, and Luminal B. Based on the existing studies, TNBC and Basal can easily be regarded as the same subtype (Wiese et al., 2013). We obtained BCII with 178 samples (Supplementary Fig.1.B).

Finally, we conducted an experiment using a gastric cancer data set, numbered E-GEOD-35809 (GC, https://www.ebi.ac.uk/arrayexpress/experiments/E-GEOD-35809/). Gastric cancer is also a common malignant cancer (Crew and Neugut, 2006). Its incidence rate remains high in the global incidence statistics of malignant cancers (Hartgrink et al., 2009). In addition, the existing studies have found that gastric cancer also has multiple subtypes. The data set used in this experiment includes three subtypes: proliferative, invasive, and metabolic (Supplementary Figure 1C) (Lei et al., 2013; Zeng et al., 2018). The purpose of using gastric cancer data is to test whether the DM-ESPCA model can be applied to different cancer subtypes’ research.

In this study, we used a mixed model of GC-RMA to preprocess all these three data sets to reduce the negative impact of the batch. Specifically, we discarded all the probes with a log2 intensity of less than 4.

### Gene Pathway Data Sets

The basic network data set used by the DM-ESPCA model is obtained from the following database: Pathway Commons database (http://www.pathwaycommons.org/).

Totally, the BCI and BCII data sets retained the same 29,873 gene probes, and the corresponding relationship network retained 1,239,154 edges. The GC data set retained 28,838 gene probes, and 1,181,312 edges were retained in the corresponding relationship network.

### Methods

In this section, we first introduced the general sparse PCA framework (SPCA). Then, we introduced the ESPCA model. Finally, we proposed the DM-ESPCA model which includes meta-data selection, the dynamic gene network, and the random sampling algorithm based on the greedy principle.

#### SPCA

Suppose there is a gene matrix $X\in R^{m,n}$ containing m genes and n samples. Using the $L_{0}$ norm for sparseness, we can get the following expression matrix (Yuan and Zhang, 2013):

$$\underset{∥u_{2}∥\leq 1}{maximize}u^{T}XX^{T}u,\;s.t.\;∥u_{0}∥\leq s$$ (1)

where u is the m×1 vector to represent the first principal component (PC) loading and s represents the number of genes retained by the model, and $u_{2}$ and $u_{0}$ represent the $L_{2}$ and $L_{0}$ norms, respectively. Researchers usually use the SVD framework to solve this problem (Lin et al., 2016). Therefore, the formula can also be written as

$$\underset{{\left\|u\right\|}_{2}\leq 1,{\left\|v\right\|}_{2}\leq 1}{maximize}u^{T}Xv,\;s.t.∥u{∥}_{0}\leq s$$ (2)

where v is n×1 PC. The problem is solved using the following strategies:

$$\mathrm{u\leftarrow}\frac{\hat{u}}{\left\|\hat{u}\right\|},\;\mathrm{where}\;\hat{u}\mathrm{=P}\left(\mathrm{z,s}\right)\;\mathrm{and}\;\mathrm{z=Xv}$$ (3)

$$v\leftarrow \frac{\hat{v}}{\left\|v\right\|},where\;\hat{v}=X^{T}u$$ (4)

where P(z,s) represents sparse projection. In the vector u , its k -th element has the following defined:

$${\left[P\left(\mathrm{z,s}\right)\right]}_{k}=\{\begin{array}{c} z_{k}\mathrm{, if k}\in \mathrm{supp}\left(\mathrm{z,s}\right)\\ \mathrm{0,otherwise} \end{array}$$ (5)

where supp(z,s) denotes the set of indexes of the largest s absolute element of z .

#### ESPCA

In 2018, Min et al. proposed the edge group sparse PCA (ESPCA), which uses known genome structures as prior knowledge (Min et al., 2018). The ESPCA model is transformed from a traditional point sparse to a group sparse which effectively improves the feature screening ability of sparse PCA. Suppose G is a group structure, in the gene interaction network, the two linked genes can be considered as a group. Obviously, such edge groups are overlapping. We denoted $G=\left\{e_{1},\ldots ,e_{m}\right\}$ as an edge set with all edges from a given gene interaction network. Here, the ESPCA model is as follows:

$$∥{\mathrm{u∥}}_{ES}=\underset{\forall G^{\prime }\in G,support\left(u\right)\subseteq V\left(G^{\prime }\right)}{minimize}\left|G^{\prime }\right|$$ (6)

where $G^{\prime }$ is a subset of G , $V\left(G^{\prime }\right)$ is a vertex (gene) set induced from the edge set $G^{\prime }$ , $\;\left|G^{\prime }\right|$ denotes the number of elements of $G^{\prime }$ , and support(u) denotes the set of indexes of the non-zero elements of u (Min et al., 2018). Based on formula 6, this sparse model can be expressed as the following formula:

$$\underset{{\left\|u\right\|}_{2}\leq 1,{\left\|v\right\|}_{2}\leq 1}{maximize}u^{T}Xv,\;s.t.∥u{∥}_{ES}\leq s$$ (7)

where s is the amount of edges. The model is solved based on a greedy algorithm.

#### DM-ESPCA

**FIGURE 2:**
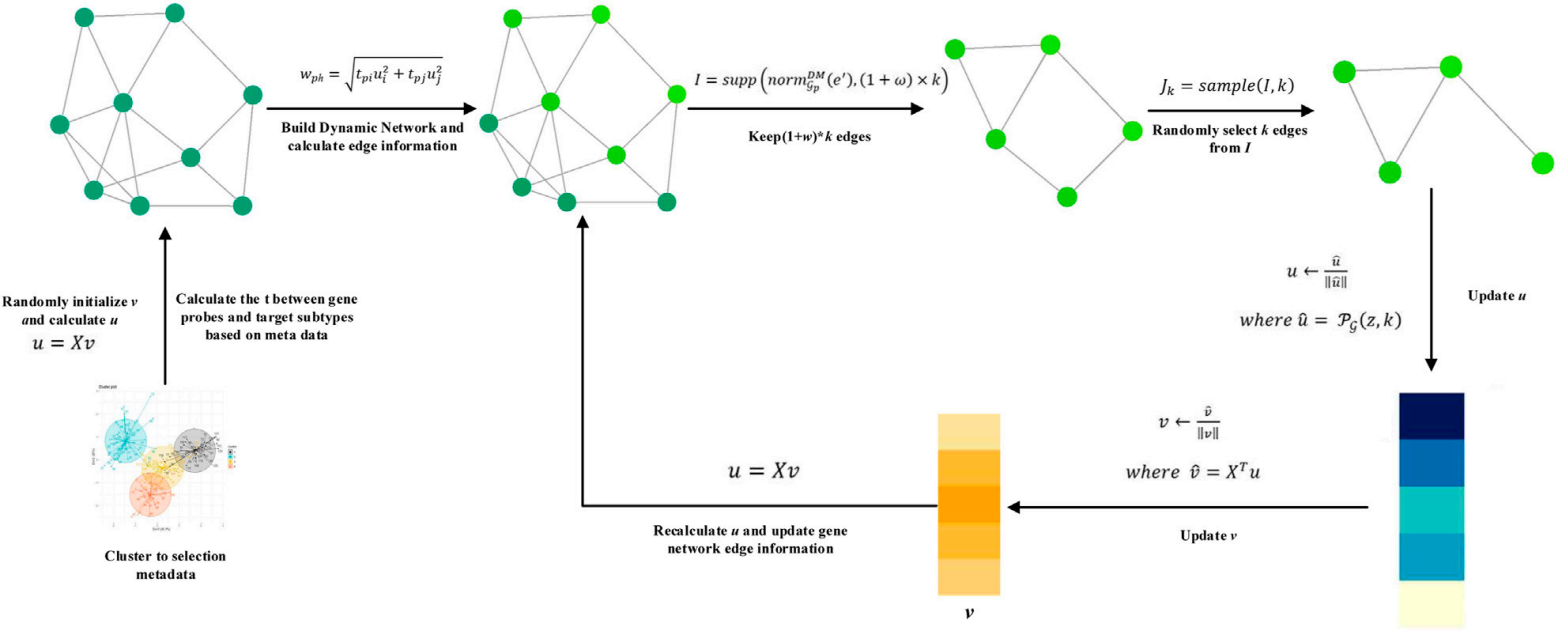
Algorithm of the DM-ESPCA model.

On the basis of SPCA and ESPCA models, we propose the DM-ESPCA model. Compared to existing models, the DM-ESPCA model has three main improvements. First, the DM-ESPCA model generates independent dynamic network weights for each PC based on known cancer subtype classification information and integrates the weights into the sparse PCA framework which enhances the model’s cancer subtype target selection capabilities. Second, in the process of generating the dynamic network weights of the DM-ESPCA model, the DM-ESPCA model improves the sample quality and noise of the data set by selecting a subset of meta-data. It ensures the accuracy and reliability of the dynamic network weights. Third, the DM-ESPCA model improves the traditional greedy algorithm and proposes a random sampling algorithm based on the greedy principle, which improves the local optimal solution of the model. Next, we introduce the details of meta-data selection, the dynamic network, and the random sampling algorithm based on the greedy principle modules in the order of model construction (Figure 2).

##### Meta-data Selection

The cancer subtype data sets are inevitably noisy, which will mislead the results of machine learning models (since the cancer subtype data sets are inevitably noisy and mislead the results of machine learning models). To solve this problem, the establishment of the dynamic network is based on meta-data (high-quality samples) after preprocessing, not all samples. Here, we adopt the idea of meta-learning to initialize model parameters with high-quality samples as much as possible and guide the operation of the entire model. It should be stated that the idea of meta-learning here means that the model uses a batch of high-quality sample data sets to guide the training of the model based on all samples (Shu et al., 2019). It does not refer to the multi-task meta-learning training mode similar to the MAML model (Finn et al., 2017). The following content is the steps for selecting meta-data from the cancer subtype data set:

First, we use all gene probes to cluster the subtype data sets which adopt the K-means algorithm.

According to the known clustering information, we select h samples closest to the cluster center point in each cancer subtype.

We repeat clustering multiple times, and the final result is that the samples are stably selected each time.

##### Dynamic Meta-data Network

Existing sparse PCA methods are all reference-free methods. Even in the ESPCA model, its used weights of the biological networks for the principal components are the same. In this article, we pre-calculate the correlation weights of each gene probe and each cancer subtype based on general biological knowledge and meta-data. These weights are used to establish a dynamic biological network for each cancer subtype, thereby enhancing the model’s gene screening ability.

Here, we presented the DM-ESPCA model as formulas 8 and 12. First, we assume that $e_{h}=\left(u_{i},u_{j}\right)\in \;G,u_{i},u_{j}\in R^{m}$ , and the weight $w_{h}\;$ of $e_{h}$ is defined as formula 8:

$$w_{h}=\sqrt{u_{i}^{2}+u_{j}^{2}}$$ (8)

where $u_{i}$ and $u_{j}$ are the left and right gene probes of $e_{h}$ , respectively.

Then, we adopted formula 9 to pre-calculate the correlation weight $t_{pi}\;$ of p−th subtype and i -th gene probe in the dynamic network of the DM-ESPCA model

$$t_{pi}=\frac{\left(x_{i}-x_{-i}\right)}{\sqrt{\frac{s_{i}^{2}}{n_{i}}+\frac{s_{-i}^{2}}{n_{-i}}}}$$ (9)

where $x_{\text{i}}$ and $s_{\text{i}}$ are the average value and the standard deviation of the i -th gene probe in the p -th subtype with meta-data samples, respectively, and $n_{\text{i}}$ is the number of samples of the p -th subtype in the meta-data. $x_{-\text{i}}$ and $s_{-\text{i}}\;$ indicate the average value and the standard deviation of the samples with the i -th gene probe not in the p -th subtype, respectively, and $n_{-\text{i}}$ represents the number of the samples not in the p -th subtype.

Therefore, the weight of the i -th gene probe in p -th subtypes in the dynamic gene network can be expressed as

$$w_{ph}=\sqrt{t_{pi}u_{i}^{2}+t_{pj}u_{j}^{2}}$$ (10)

Here, the dynamic network of the p -th subtype can be represented as $G_{p}={\left\{w_{\text{ph}}e_{\text{h}}\right\}}_{1}^{m}$ . According to formula (10), we can construct a completely different gene network for each cancer subtype. Our purpose of constructing the dynamic network is to hope that the DM-ESPCA model screens the gene probes which are most relevant to the corresponding cancer subtype. Then, we can use the following dynamic meta-data (DM) network as the sparse penalty:

$${\mathrm{∥u∥}}_{\mathrm{DM}}=\underset{\forall G_{p}^{\prime }\in G_{p}\mathrm{,support}\left(u\right)\subseteq V\left(G_{p}^{\prime }\right)}{\mathrm{minimize}}\left|G_{p}^{\prime }\right|$$ (11)

where $G_{p}^{\prime }$ is a subset of $G_{p}$ , $V\left(G_{p}^{\prime }\right)$ is a vertex (gene) set induced from the edge set $G_{p}^{\prime }$ , $\;\left|G_{p}^{\prime }\right|$ denotes the number of elements of $G_{p}^{\prime }$ , and support(u) denotes the set of indexes of nonzero elements of u .

Finally, the sparse model of this article can be represented as

$$\underset{{\left\|u\right\|}_{2}\leq 1,{\left\|v\right\|}_{2}\leq 1}{maximize}u^{T}Xv,\;s.t.∥u{∥}_{DM}\leq k$$ (12)

where u is the first PC loading, v is the first PC, and k is the parameter to control the number of edges selected for each cancer subtype.

##### Random Sampling Algorithm Based on the Greedy Principle

To solve sparse PCA methods, the key issue is how to solve a projection problem with fixed v and z (z=Xv) . This is a typical NP-hard problem (Min et al., 2018). Many of the traditional sparse PCA methods use $L_{0}$ and the greedy principle to screen the gene probes with the largest weights. However, the greedy principle will mislead a local optimal solution. Here, we proposed a random sampling algorithm based on the greedy principle to find a better solution of the DM-ESPCA model. We adopted the idea of a simulated annealing algorithm and add randomization to the traditional greedy algorithm. Existing research shows that introducing randomization parameters into the model can improve the local optimal solution problem of the greedy algorithm (Van Laarhoven and Aarts, 1987; Rutenbar, 1989). In addition, due to the difficulty of convergence caused by randomization parameters, we also designed an independent parameter to reduce the randomization rate during the model cycle and finally reduce the randomization rate to 0 to ensure that the model can converge. Note that we cannot guarantee that the algorithm converges to the optimal solution due to the non-convexity of this problem. Thus, we repeated our algorithm with a number of different random initial solutions.

In algorithm 1, $P_{G}\left(\mathrm{z,k}\right)$ is the sparse projection; $\;{\left[P_{G_{p}}\left(\mathrm{z,k}\right)\right]}_{i}\left(\mathrm{i=1,\ldots ,m}\right)$ meets

$${\left[P_{G_{p}}\left(\mathrm{z,k}\right)\right]}_{i}=\{\begin{array}{c} z_{i},\;\mathrm{if}\;G_{p}\left(i\right)\cap \mathrm{sample}\left(\mathrm{I,k}\right)\neq \emptyset \\ \mathrm{0,otherwise} \end{array}$$ (13)

where $G_{p}\left(i\right)$ is the edge set of the gene network corresponding to the cancer subtype p and $I=supp\left(norm_{G_{p}}^{DM}\left(e^{\prime }\right),\;\left(1+\omega \right)\times k\right)$ . If gene i is selected, ${\left[P_{G}\left(\mathrm{z,k}\right)\right]}_{i}=z_{i}$ ; otherwise, ${\left[P_{G}\left(z,k\right)\right]}_{i}=0$ . k represents the number of edges expected to be retained. ω is a parameter that controls the random ratio. For example, if we set the parameter k=100 , ω=0.2 , then the algorithm will keep 120 edges with the largest weight in each cycle and randomly select 100 of them as the result.

Finally, we use formulas 14, 15 to update vectors u and v until the algorithm convergence:

u=Xv

$$\mathrm{where}\;\hat{v}=X^{T}u$$

$$\mathrm{u\leftarrow}\frac{\hat{u}}{\left\|\hat{u}\right\|},\;\mathrm{where}\;\hat{u}=P_{G}\left(\mathrm{z,k}\right)\;\mathrm{and}\;\mathrm{z=Xv}$$ (14)

$$v\leftarrow \frac{\hat{v}}{\left\|\hat{v}\right\|}\mathrm{,where}\;\hat{v}=X^{T}u$$ (15)

Algorithm 1 Random sampling algorithm based on the greedy principle sparse projection for the dynamic network

$$\mathrm{Require: X}\in R^{\mathrm{m\times n}}\mathrm{,\nu}\in R^{\mathrm{n\times 1}}\mathrm{,parameter}\;\mathrm{k,\omega ,\rho ,}$$

$$\mathrm{edge}\;\mathrm{set}\;G_{P}=\left\{e_{1},e_{2}\mathrm{,L}e_{n}\right\}$$

1:Z=Xν

2:for any weight of edge e in GP do

$$\mathrm{3:}w_{n}^{\prime }=\sqrt{t_{\mathrm{Pi}}{Ζ}_{i}^{2}+t_{\mathrm{Pj}}{Ζ}_{j}^{2}}\;\mathrm{\#Generate}\;a\;\mathrm{dynamic}\;\mathrm{network}.$$

4:update GPn′= wn′

5:end for

$$\mathrm{6:Let}\;\mathrm{nor}m_{G_{P}^{\prime }}^{\mathrm{DM}}\left(e^{\prime }\right)={\left(\left\|e_{1}^{\prime }\right\|\mathrm{,\ldots ,}\left\|e_{n}^{\prime }\right\|\right)}^{T}$$

$$\mathrm{7:I=supp}\left(\mathrm{nor}m_{G_{p}}^{\mathrm{DM}}\left(e^{\prime }\right),\left(\mathrm{1+\omega}\right)\mathrm{\times k}\right)\mathrm{\#Extract}\left(\mathrm{1+\omega}\right)\mathrm{\times k}\;\mathrm{edges}.$$

$$\mathrm{8:}J_{k}\mathrm{=sample}\left(\mathrm{I,k}\right)\;\mathrm{\#Randomly}\;\mathrm{select}\;k\;\mathrm{edges}\;\mathrm{from}\;I.$$

9:if  ω>0 then  ω= ω-ρ #Reduce random rate

$$\mathrm{10:}V_{\;G_{P}^{\prime }}\mathrm{=V}\left(G_{P}^{\prime }\right)$$

$$\mathrm{11:for}\;\mathrm{any}\;\mathrm{gene}\;i\;\mathrm{in}\;V_{\;G_{P}^{\prime }}\;\mathrm{do}$$

$$\mathrm{12:}{\hat{u}}_{i}=z_{i}$$

13:end for

$$\mathrm{14:u=}\frac{\hat{u}}{\left\|\hat{u}\right\|}$$

$$15:\mathrm{return}\;u\;and\;P_{G_{P}}\left(z,k\right)=\hat{u}$$

In order to ensure the convergence of the algorithm, when the model completes the edge sparse projection, we use the parameter  ρ to reduce the randomness of the model, that is, if ω>0, ω=ω− ρ . Furthermore, the DM-ESPCA model can be applied to generate multiple PCs and their PC loadings. Specifically, given the current PCs, we adopted Min’s model to compute the next PC and its loading (Min et al., 2018).

## Results

The experiments are divided into two steps. First, we use three sparse PCA methods including DM-ESPCA, ESPCA, and SPCA models to perform unsupervised sparse PCA on the cancer data sets. This step will allow each model to screen the subset of the potential target genes for each cancer subtype. We adopted three indicators including heat map, the cluster results, and *p*-value to evaluate the gene subset screen by each model. We also conducted a bio-enrichment analysis (Zhou et al., 2019) to count the key biological pathways corresponding to these gene subsets, such as the GO biological process (GO-BP), KEGG, and so forth, to determine whether these gene subsets are related to the cancer subtypes.

In order to further compare these gene subsets screened by the three sparse PCA methods, we used all samples based on the gene subsets to build four machine learning classification models, such as the K-Nearest Neighbor (KNN) model, the Support Vector Machines (SVM), the Logistic Regression, and the Random Forest model (Hearst et al., 1998; Liaw and Wiener, 2002; Peterson, 2009). In addition, we also built four machine learning models based on all genes, which was performed to compare whether the DM-ESPCA model is better than the classic supervised learning model in classification tasks. In sections 3.1–3.3, we only illustrate the results of the KNN model, and the results of other models are in the supplementary materials. Four classic statistical indicators, including precision, recall, F1-score, and accuracy, are used to evaluate the classification results. All machine learning experiments use the 5-fold cross-validation approach, and the final results are the averages of five runs. (The detail of indicators is in the Supplementary Materials.)

### Application to the BCI Data Set

**FIGURE 3:**
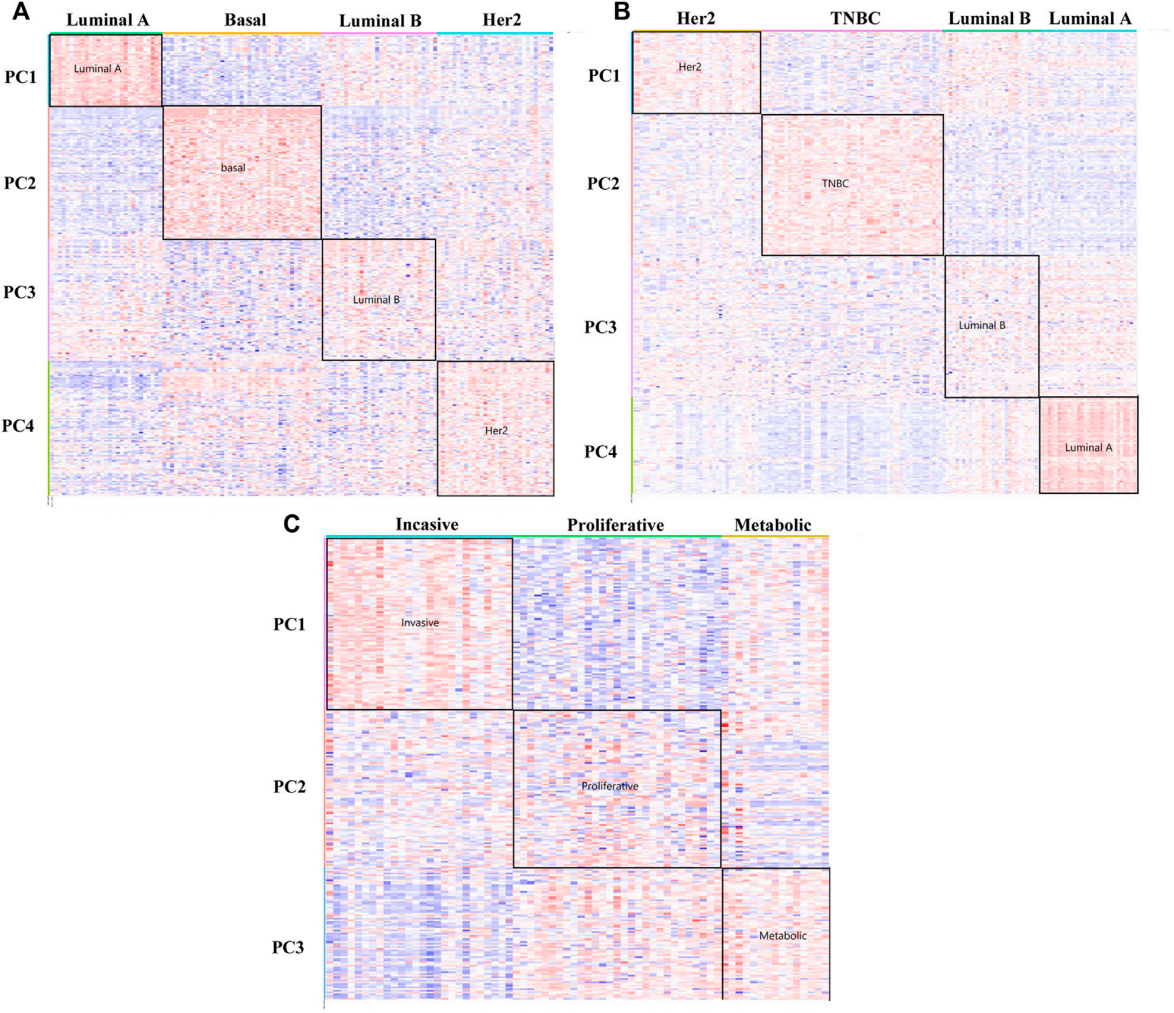
Heat maps of the DM-ESPCA model. **(A)** Result of the BCI data set. **(B)** Result of the BCII data set. **(C)** Result of the GC data set. The row is the gene probs; different color blocks of rows indicate genes selected by different PC loadings. The column is the samples. The color of each block in the heat maps is the expression value of the genes.

In Figure 3A of the heat map analysis, we can find that the DM-ESPCA model can clearly distinguish the four breast cancer subtypes with clear boundaries. However, the gene probes screened by the ESPCA and SPCA models could not distinguish these four subtypes well (Supplementary Figure S2, S3). Table 2 summarizes these clustering results, where the clustering accuracy of the DM-ESPCA model reached 82.3%, which is 14.61% higher than the results of the ESPCA model and 21.6% higher than that of the results of the SPCA model (Supplementary Table S1). These results showed that the DM-ESPCA model had a relatively strong distinguishing ability for the four subtypes of breast cancer, especially in Luminal B subtypes. In addition, according to the *p*-values shown in Figure 7A and Supplementary Figure S10, the performance of the DM-ESPCA model was significantly better than that of the ESPCA and SPCA models in the correlation of Luminal A subtype. Moreover, the average *p*-values of select genes in all subtypes are very low, which means that the results of our proposed model are highly related to breast cancer (Supplementary Table S2).

**TABLE 2:** Clustering results obtained by the three sparse PCA methods.

	DM-ESPCA (%)	ESPCA (%)	SPCA (%)
BCI	**82.30**	67.69	60.70
BCII	**82.35**	75.16	59.87
GC	**82.86**	77.14	78.57

**FIGURE 4:**
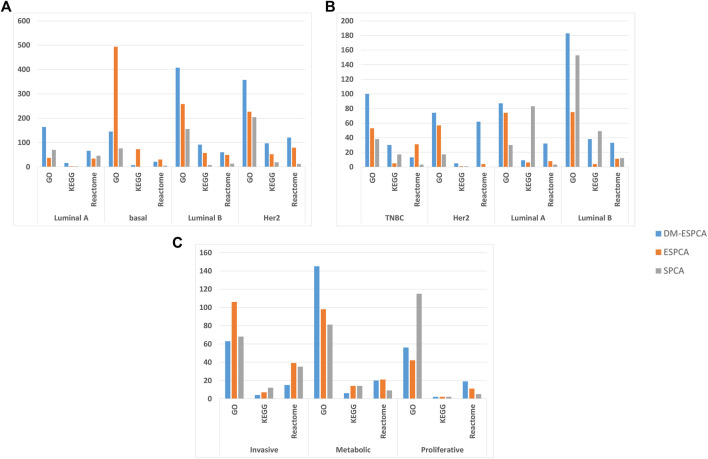
Pathway numbers with screened genes of GO, KEGG, and Reactome in the bio-enrichment analysis; **(A)** number of pathways in the BCI data set; **(B)** number of pathways in the BCII data set; **(C)** number of pathways in the GC data set. The blue bar is the DM-ESPCA model, the orange bar is the ESPCA model, and the gray one is the SPCA model.

**FIGURE 5:**
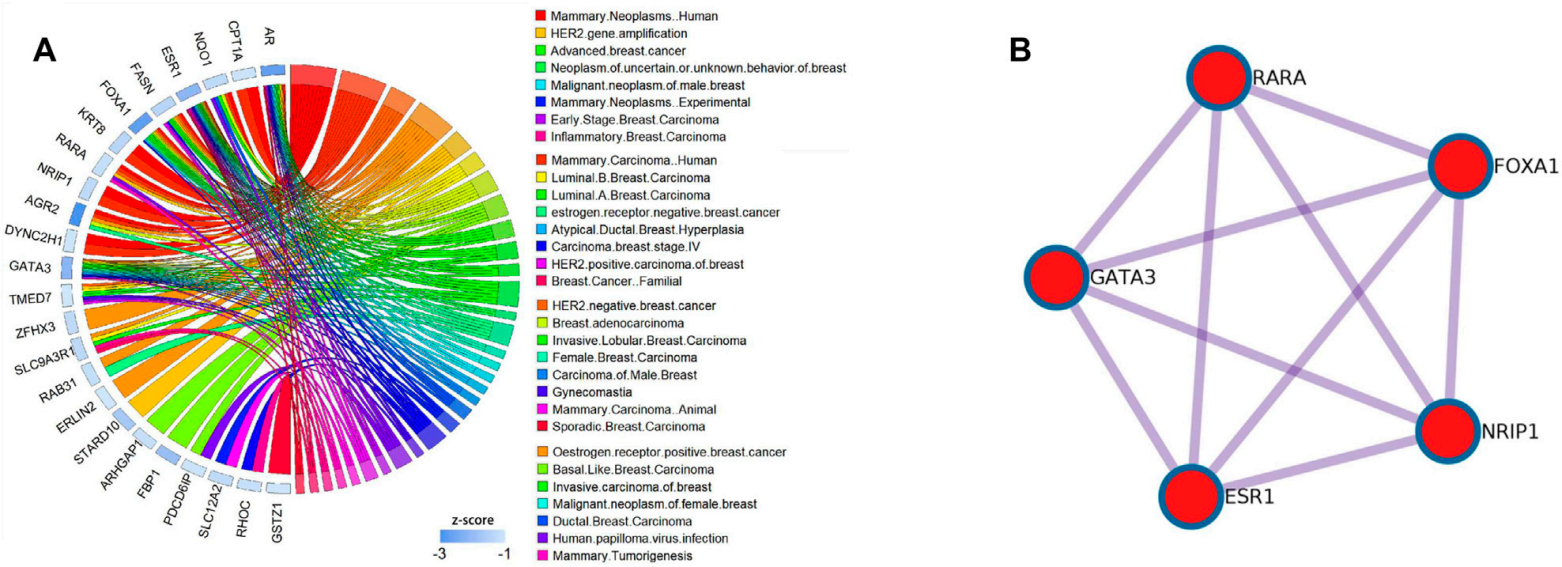
Results of the DisGeNET dataset and PPI pathways of the Basal subtype in the BCI dataset; **(A)** relationship between the diseases and gene selected by the DM-ESPCA model of the Basal subtype in the BCI dataset.The blue bar shows the z-score of each gene.Data collected from the DisGeNET dataset. **(B)** KeyPPI pathways of part of the gene selected by the DM-ESPCA data set.

In order to further verify the gene screening ability of the DM-ESPCA model, we conducted a bio-enrichment analysis. It can be seen from Table 3 that the DM-ESPCA model can find genes related to breast cancer in all four subtypes, but the ESPCA and SPCA models can only be found in three subtypes. From Figures 4, 5, we can see that the DM-ESPCA model can find 1,286 biological pathways in the GO-BP and KEGG data sets. These results are much better than that of the ESPCA and SPCA models.

**TABLE 3:** Number of PCs that can find gene probes related to the target cancer for each model.

	DM-ESPCA	ESPCA	SPCA
BCI	4	3	3
BCII	4	2	3
GC	3	0	0

**FIGURE 6:**
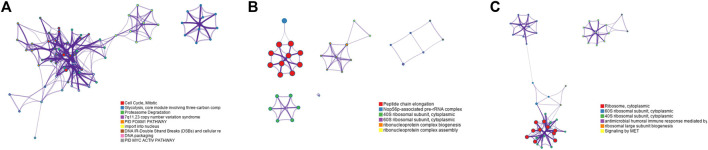
Functional pathways collected from the BCI data set Luminal A subtype; **(A)** results of GO-BP in the DMESPCA model; **(B)** results of GO-BP in the ESPCA model; and **(C)** results of GO-BP in the SPCA model.

Among the results of the enrichment analysis, the basal subtype results of the DM-ESPCA model are particularly encouraging. First, in the PPI networks, it found multiple key target protein sites. Among them, ESR1, NRIP1, FOXA1, RARA, and GATA3 are highly correlated with the gene pathway R-HSA-9018519 of estrogen-dependent gene expression (Figure 6B). The secretion of estrogen is one of the important causes of breast cancer. We also found that the z-scores of the aforementioned gene probes are generally high (Supplementary Figure S8). Next, in the DisGeNET set, the potential target gene probes screened by the DM-ESPCA model are related to 32 known breast cancer disease signatures (Figure 6A). Among them, the gene probes ARHGAP1, ESR1, FBP1, GATA3, FOXA1, PDCD6IP, AR, FASN, RARA, and TMED7 are directly related to the basal-like breast carcinoma and HER2-negative breast cancer with the data set numbers C3642347 and C4733095 (Supplementary Table S3). Finally, the enrichment analysis results of the PaGenBase data set show that the gene set found by the DM-ESPCA model is highly correlated with breast cells (Supplementary Table S4). In general, the results of the gene enrichment analysis clearly prove that DM-ESPCA has a strong ability to select target genes of breast cancer subtypes.

**FIGURE 7:**
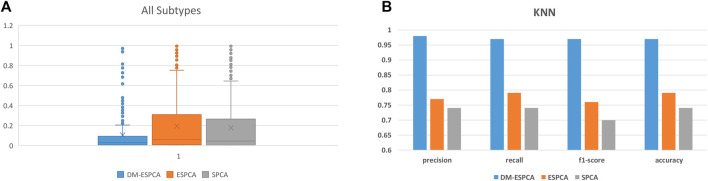
Boxplots and classification comprehensive indicators of the BCI data set; **(A)** *p*-values of selected genes in all subtypes. **(B)** Results of KNN in three sparse PCA methods and the use of all genes.

The gene subset selected by the DM-ESPCA model also achieved the best classification results; the accuracy reached 97%, the precision reached 98%, the recall reached 97%, and the F1-score reached 97% (Figure 7B, Supplementary Table S5). Simultaneously, the classification accuracy based on the gene subset selected by the ESPCA model and its precision, recall, and F1-score only reached 77, 79, 76, and 76%, respectively. The classification accuracy based on the gene subset selected by the SPCA model and its precision, recall, and F1-score only reached 75, 74, 71, and 74%, respectively. It is worth noting that even if we use all genes to build four supervised machine learning models, the best result of precision, recall, and F1-score only reached 85, 86, 85, and 85% (Logistic Regression model), which is much lower than the result of the DM-ESPCA model.

In summary, these results demonstrated that the DM-ESPCA model can identify more biologically relevant gene sets than the ESPCA and SPCA models. In classification tasks, the DM-ESPCA model is better than ESPCA, SPCA, and classic supervised learning models. From the perspective of model construction, it is expected that the DM-ESPCA model can obtain better results than ESPCA and SPCA in heat map, cluster analysis, correlation analysis, enrichment analysis, and classification experiments. Because the dynamic network takes known cancer subtype classification information as prior knowledge, this enables the DM-ESPCA model to select cancer targets that are more relevant to the corresponding cancer subtype. The screening of meta-data further alleviates the problem of sample quality in the data, and the random sampling algorithm based on the greedy principle improves the local optimal solution problem of the traditional greedy algorithm. In addition, we believe that the dimensional challenges and overfitting problems of the data prevent the machine learning model (use all gene probes) from achieving a better performance, which is the same point of view as existing research works.

### Application to the BCII Data Set

In order to further verify the stability of the DM-ESPCA model in the same type but different batches of cancer subtype data sets, we also used the BCII data set to conduct the experiments, which showed similar results compared with the BCI data set. According to Figure 3B, the DM-ESPCA model could distinguish four breast cancer subtypes well, and the boundary corresponding to each subtype was very clear. In contrast, the heat map results of the ESPCA and SPCA models were worse in the BCII data set, and they were difficult to judge the boundary of the subtype (Supplementary Figure S4, S5). In Table 2, the cluster accuracy of the DM-ESPCA model reached 82.3%; however, the cluster accuracies of the ESPCA and SPCA models only reached 75.1 and 59.8%, respectively. Similar to the results in the BCI data set, the Lumina B subtype was difficult to distinguish; the DM-ESPCA model could relatively accurately divide all samples into four subtypes, including the Lumina B subtype. Neither the ESPCA model nor the SPCA model could cluster Lumina B subtypes well (Supplementary Table S6). Besides, in Supplementary Fig.11, the DM-ESPCA model outperformed the ESPCA and the SPCA models in *p*-values, especially the correlation of a comprehensive Luminal A subtype (Supplementary Table S7). These meant that the genetic points screened by the DM-ESPCA model had a higher correlation with cancer subtypes, which was more conducive to the analysis by biological researchers.

An enrichment analysis showed that the DM-ESPCA model selected gene probes containing the largest number of biological pathways (Figure 4B). In addition, the DM-ESPCA model can find gene probes known to be related to breast cancer diseases in the DisGeNET set among all four principal components (Table 3). In comparison, the ESPCA model can only find genes related to breast cancer in two principal components, while the SPCA model can find genes related to breast cancer in three principal components. Especially in the Luminal B subtype, the DM-ESPCA model can find 13 gene probes related to eight breast cancer disease entries which show a very high correlation with breast cancer (Supplementary Figure S9).

Finally, based on Supplementary Fig.12, the optimal classification results were obtained by the KNN method based on the gene subset selected by the DM-ESPCA model. Its accuracy, precision, recall, and F1-score reached 90, 90, 89, and 88%, respectively. In comparison, these four classification indicators of the model based on the gene subset selected by the ESPCA model could only reach 86, 86, 80, and 80%, respectively, while these four classification indicators of the model based on the gene subset selected by the SPCA model could only reach 82, 82, 80, and 80%, respectively (Supplementary Table S8). The best results of precision, recall, and F1-score for the supervised machine learning model which used all genes only reached 85, 87, 85, and 85% (Logistic Regression model), which is lower than the result of the DM-ESPCA model, 5, 3, 4, and 3%.

Based on the results of the BCII data set, we can see that in the same cancer subtype, but in different data batches, the performance of the DM-ESPCA model was very stable.

### Application to the GC DataSet

To verify the applicability of the DM-ESPCA model in different cancer data, we used a gastric cancer data set for experimentation. Based on the result of the heat map (Figure 3C, Supplementary Figure S6, S7), the DM-ESPCA model performed well, especially in subtypes Invasive and Metabolic. In Table 1, the clustering accuracy of the DM-ESPCA model reached 84.23%. Compared with the ESPCA and SPCA models, the clustering accuracy of the DM-ESPCA model increased by 9 and 6%, respectively (Supplementary Table S9). Meanwhile, based on Supplementary Fig.13, the *p*-values of the DM-ESPCA model have had significant improvements compared with other models (Supplementary Table S10).

In addition, it can be seen from Figure 3C, the DM-ESPCA model has more number of GO, KEGG, and Reactome pathways than the comparison methods in bio-enrichment analysis. In particular, the DM-ESPCA model is the only one that can find genetic probes related to all subtypes of gastric cancer. However, neither ESPCA nor SPCA can find genes related to gastric cancer in the three subtypes (Table 3).

Based on Supplementary Fig.14, the optimal classification results were obtained by the KNN method based on the gene subset selected by the DM-ESPCA model. Its accuracy, precision, recall, and F1-score reached 95, 96, 95, and 95%, respectively. In comparison, these four classification indicators of the model based on the gene subset selected by the ESPCA model could only reach 76, 72, 77, and 73%, respectively. While these four classification indicators of the model based on the gene subset selected by the SPCA model could only reach 86, 86, 86, and 86%, respectively (Supplementary Table S11). The best results of precision, recall, and F1-score for the supervised machine learning model which used all genes only reached 90, 93, 90, and 91%, respectively (Logistic Regression model), which is also lower than the result of the DM-ESPCA model. In summary, whether in the same cancer data sets with different batches or in different cancer data sets, the DM-ESPCA model performed better than the existing sparse PCA methods. Therefore, we believe that the DM-ESPCA model could reliably and stably screen the gene probes corresponding to the cancer subtypes.

### Ablation Experiment

In order to further verify the influence of three main modules of DM-ESPCA, which include the random sampling algorithm based on the greedy principle, the dynamic network, and the meta-data selection module on model performance, we performed ablation experiments based on the BCI data set (Table 4, Supplementary Table S12). As shown in Table 4, non- ω refers to the experimental results with the random sampling algorithm based on the greedy principle module removed (use the greedy algorithm instead). Non-DM refers to the experimental results with dynamic network modules removed. Non-Meta refers to experimental results with meta-data selection modules removed. We use the results of clustering, accuracy, precision, recall, and F1-score as evaluation metrics. The classification experiments use the KNN method as the classifier because the KNN method performs the best on the three real data sets. The experimental results show that the three main modules proposed in this article all have a significant impact on the results. Among them, the removal of the meta-data selection module has the greatest impact on the results. After removing the meta-data, the clustering accuracy of the model dropped to 65.38% and the result of classification accuracy dropped to 79%. The experimental results mean that there are indeed sample quality issues and data noise in the data set and that it can be improved by incorporating the meta-data selection module. The dynamic network also has a great influence on the model. After removing the dynamic network module, the clustering accuracy of the DM-ESPCA model can only reach 66.15%, which shows that dynamic networks can improve model performance. In addition, the experimental results show that the random sampling algorithm based on the greedy principle can effectively improve the results of the model and alleviate the local optimal solution problem of the greedy algorithm.

**TABLE 4:** Result of the ablation experiment.

	Clustering (%)	Accuracy (%)	Recall (%)
DM-ESPCA	82.30	97	97
Non- ω	80.07	82	82
Non-DM	66.15	87	87
Non-Meta	65.38	79	78

## Discussion

Since the beginning of the 21st century, with the development of the gene sequencing technology, researchers have discovered that the same cancer can be divided into different subtypes, which also explains that the same drug is only effective for some cancer patients but not for other patients. Therefore, how to find target genes corresponding to cancer subtypes has gradually become an important task of cancer research.

The traditional screening models for potential targets of cancer subtypes have three main problems. The first problem is that no known subtype classification information can be used. In this study, we have shown that if researchers can integrate the known subtype classification information as prior knowledge to carry out cancer subtype screening models and establish a dynamic gene network, then the screening ability of potential cancer subtype targets of the model can be greatly enhanced. The second is that the experiment’s sample quality is uneven, and low-quality samples will affect the final results of analyses. In this article, we used the idea of meta-learning to screen high-quality samples. The third point is that most of the existing models adopt the greedy principle, which will make the model quickly fall into a local optimum. We designed a new random sampling algorithm to improve the model, which may find better target genes.

Based on the aforementioned ideas, this article proposes the DM-ESPCA model, which is based on meta-learning, the dynamic gene network, and sparse PCA to screen the corresponding potential target gene probes for each cancer subtype. The bio-enrichment analysis shows that the DM-ESPCA model can directly find gene probes related to the corresponding cancer subtype. Moreover, all indicators indicate that the DM-ESPCA model can reveal more modules related to biology. Even in the task of classification of cancer subtypes, the DM-ESPCA model is superior to the existing supervised learning model. In summary, we believe that the DM-ESPCA model is a good extension of the PCA-based methods. This model can provide an effective tool for researchers to find target genes corresponding to cancer subtypes.

Although the experiment has achieved good results, the DM-ESPCA model can still be extended. We have proved that the idea of meta-learning reduces the errors caused by the noise data. However, the results of the gastric cancer data set are not very satisfactory. The reason may mean that there is still noise in the meta-data. We would consider using more powerful statistical methods to filter the meta-data. In addition, the random sampling algorithm based on the greedy principle proposed in this article can also be further improved. There are many optimization principles for NP-hard problems that can be considered. This may further improve the feature selection ability of the proposed model. In addition, it is worth noting that there are many multi-omics cancer subtype target screening models. Compared with single omics, multi-omics data can provide different views of the same batch of samples, which may lead to new and interesting biological discoveries. In theory, the DM-ESPCA model can be extended to a multi-omics model. However, how to solve the multi-omics joint sparse PCA problem still needs to be further discussed.

## Data Availability

The original contributions presented in the study are included in the article/Supplementary Material, further inquiries can be directed to the corresponding author.
