# Supplementary material for: Dynamic Meta-data Network Sparse PCA for Cancer Subtype Biomarker Screening
Source: Front Genet. 2022 May 9;13:869906. doi: 10.3389/fgene.2022.869906 (PMC9197542; doi:10.3389/fgene.2022.869906)
Supplement: Supplementary file 13 [file DataSheet1.DOCX]

Supplementary Material

# Evaluation indicators

1.1 Heatmap

Heatmap can use color changes to reflect the data information in a two-dimensional matrix or table. It can intuitively express the size of the data value in a defined color shade. This article uses the gene probes obtained from each model to draw the heatmap. The purpose of using heat maps is to intuitively judge whether the gene probes screened by each model can distinguish different subtypes.

1.2 K-means

K-means model is a classic clustering algorithm based on Euclidean distance. In this paper, the K-means algorithm is executed based on the principal component information of the sample obtained by each model. We will compare the clustering results of samples with the real sample subtype results to calculate the accuracy rate.

1.3 P-value

P-value is a classic correlation analysis method. In null hypothesis significance testing, the p-value[note 1] is the probability of obtaining test results at least as extreme as the results actually observed, under the assumption that the null hypothesis is correct. A very small p-value means that such an extreme observed outcome would be very unlikely under the null hypothesis. The form of calculating P-value in this paper is as follows:

We will first perform t-test and get the T-score for each gene probe. Then, according to degrees of freedom, we use statistical software for example: Excel completes the numerical conversion to obtain the final P-values.

We calculate the P-value between the gene probes retained by each principal component and the corresponding subtype. Then we draw the box diagram. Our purpose is to determine whether the target gene screened by the model is highly correlated with the corresponding subtype. This method can more fully verify the target gene selection ability of each model.

1.4 Z-score

Z-score is the number of standard deviations by which the value of a raw score is above or below the mean value of what is being observed or measured. Raw scores above the mean have positive standard scores, while those below the mean have negative standard scores.

$$z=\frac{\mathcal{x-}\mu}{\sigma}$$

In order to further verify the target gene selection ability of the model, we also used the gene probes selected by the model to establish three machine learning models for comparison. In order to accurately evaluate the results of each model, especially considering the imbalance of the sample. We used the following four evaluation indicators:

1.5 Accuracy

Accuracy refers to the ratio of the number of correctly predicted samples to the total number of predicted samples. This is one of the most common classification indicators. However, this indicator cannot fully evaluate the model when the data is not balanced. Therefore, we also uses Precision, Recall and F1-score as comparison indicators.

1.6 Precision

Precision refers to the ratio of the number of correctly predicted positive samples to the number of all predicted positive samples.

1.7 Recall

Recall refers to the ratio of the number of correctly predicted positive samples to the total number of true positive samples.

1.8 F1-score

F1-score is equivalent to the harmonic average of precision and recall, and the result will refer to both indicators at the same time.

**2 Machine learning model**

In order to fully verify the feature selection ability of each sparse PCA model. We also use the gene probes selected by each model to build three classic machine learning models and use 5-fold cross-validation to test the models.

2.1 Support Vector Machines（SVM）

Support vector machine is a generalized linear classifier that classifies data in a supervised learning manner. The decision boundary is the maximum margin hyperplane for solving the learning sample. We uses SVM model with poly kernel function. This model has been widely used in biological data processing and has produced good results [1; 2]

2.2 Random forest

Random forest is a classic machine learning model based on decision trees. Random forest refers to a classifier that uses multiple trees to train and predict samples. This model is also a classic method of processing biological data [3].

2.3 K-NearestNeighbor （KNN）

The KNN model is one of the simplest models in machine learning. K-NearestNeighbor refers to the K nearest neighbors. The KNN algorithm believes that each sample can be represented by its K nearest neighbors. The KNN model is also widely used in biological data, especially cancer classification [4; 5].

2.4 Logistic Regression

Logistic regression is a generalized linear regression analysis model which often used in data mining, cancer classification and other fields. Logistic Regression usually uses regularization to improve the performance of the model. There are two commonly used regularizations:$L_{1}$ norm and $L_{2}$ norm. In this article, the Logistic Regression model we build adopts the $L_{2}$ norm.

**3 Parameter Setting**

Here we supplement the parameter settings not described in the paper. We set the number of k-means clustering follows number of subtypes of cancer. About 25 samples are reserved for each subtype as metadata. Due to the quality problem of the data set, the number of metadata will be adjusted. We set the random rate $\omega$ is 0.5 and the decline rate $\rho$ is 0.1. For DM-ESPCA and ESPCA, we retain $k$ edges, and $k$ equals to 100. For SPCA, we retain 100 points.

# Supplementary Figure

**
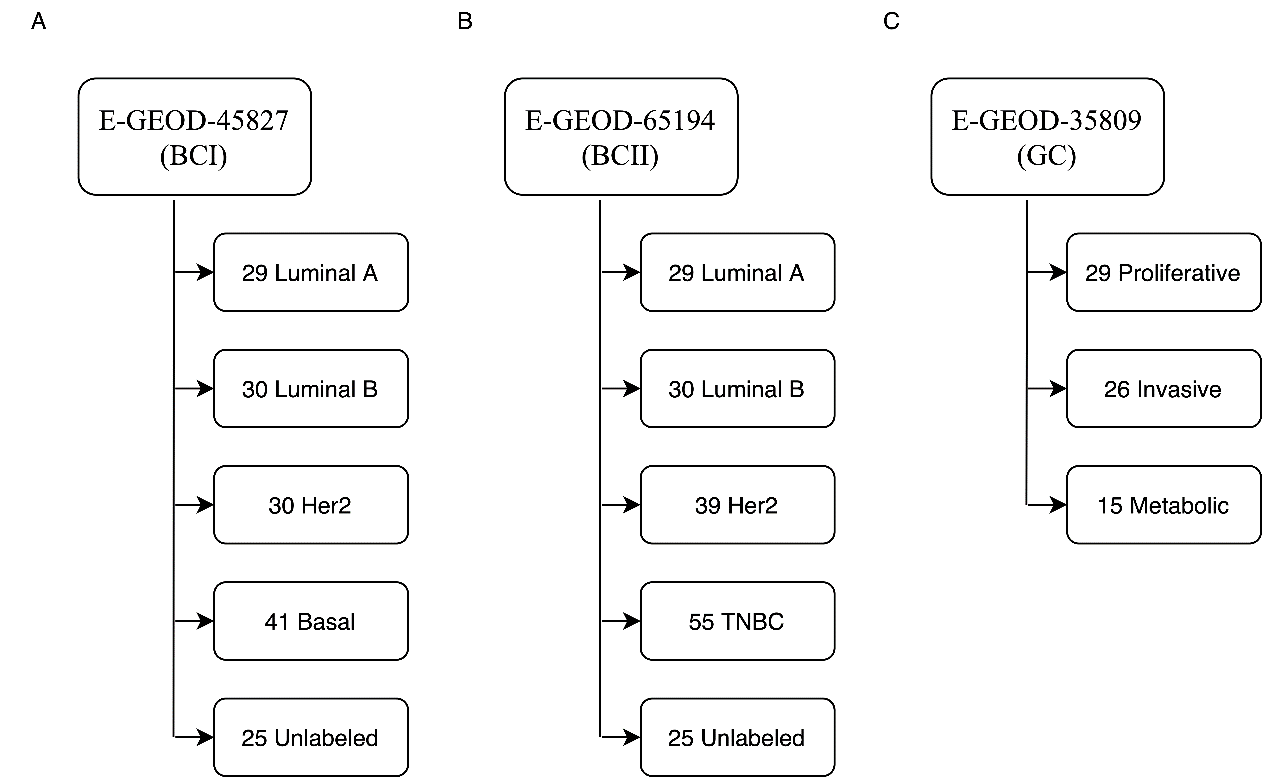
Supplementary Figure S1** Sample distribution of the three data sets.

**Supplementary Figure S2** Heatmap of BCI by ESPCA.


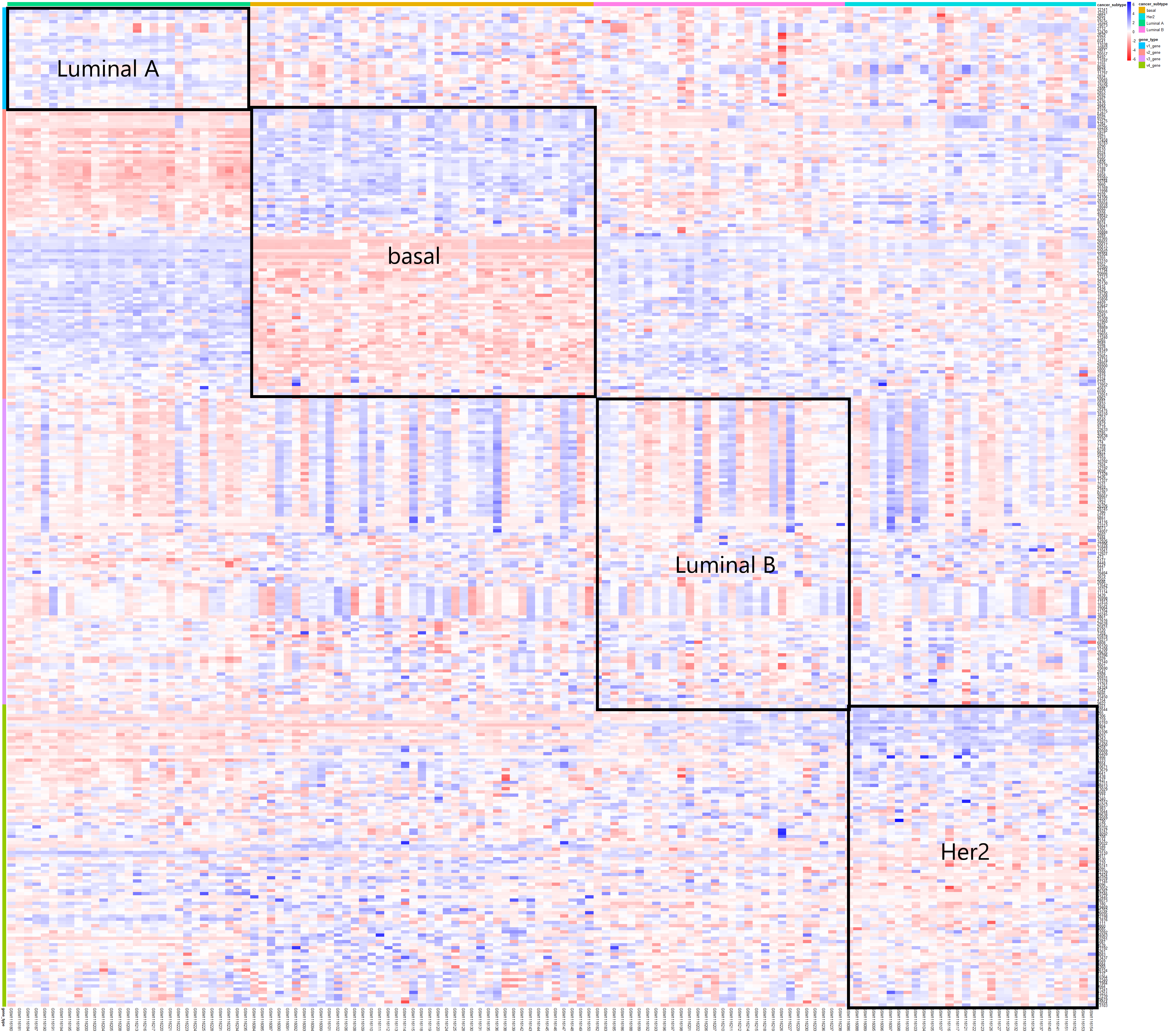


**Supplementary Figure S3** Heatmap of BCI by SPCA.


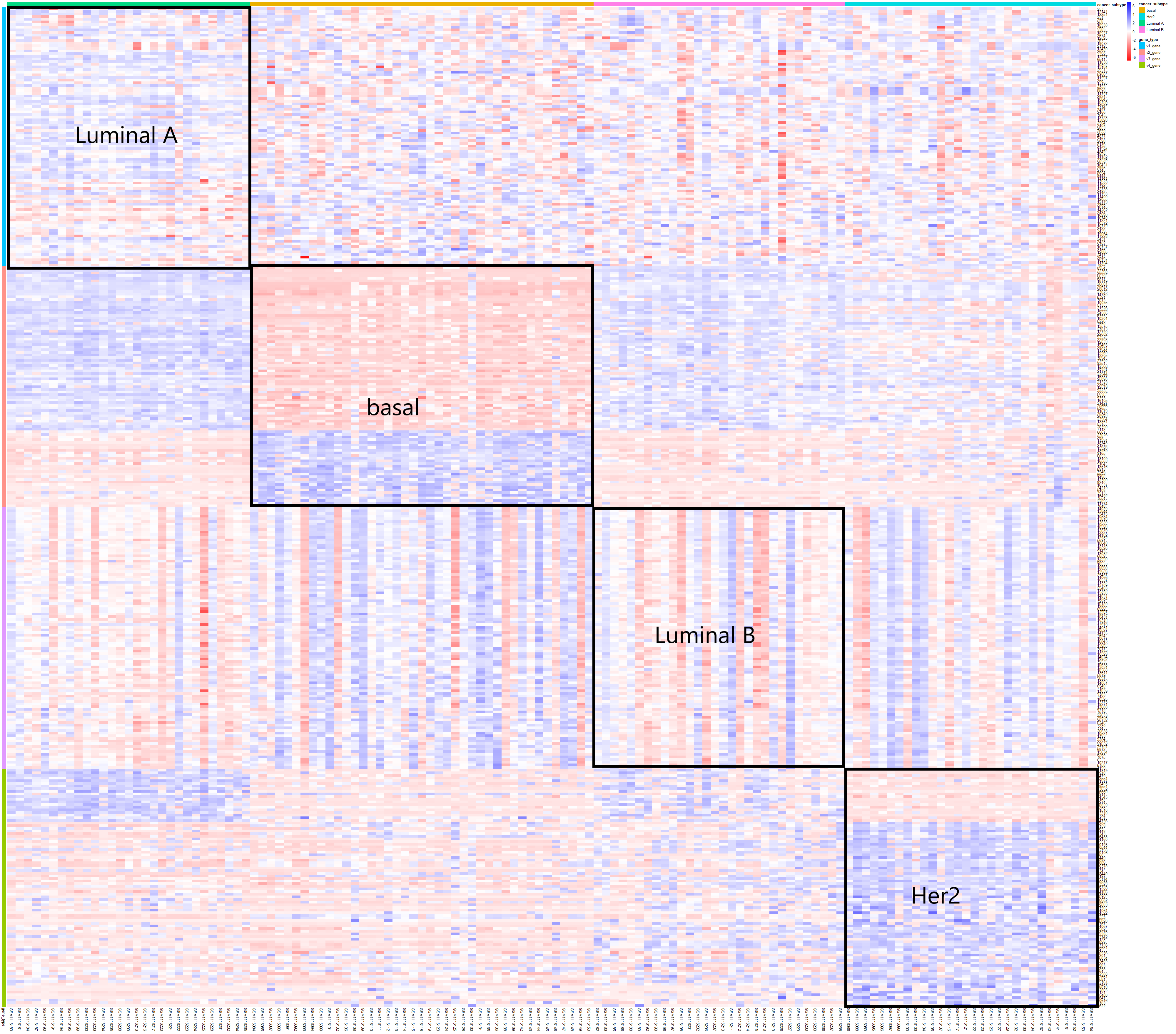


**Supplementary Figure S4** Heatmap of BCII by ESPCA.


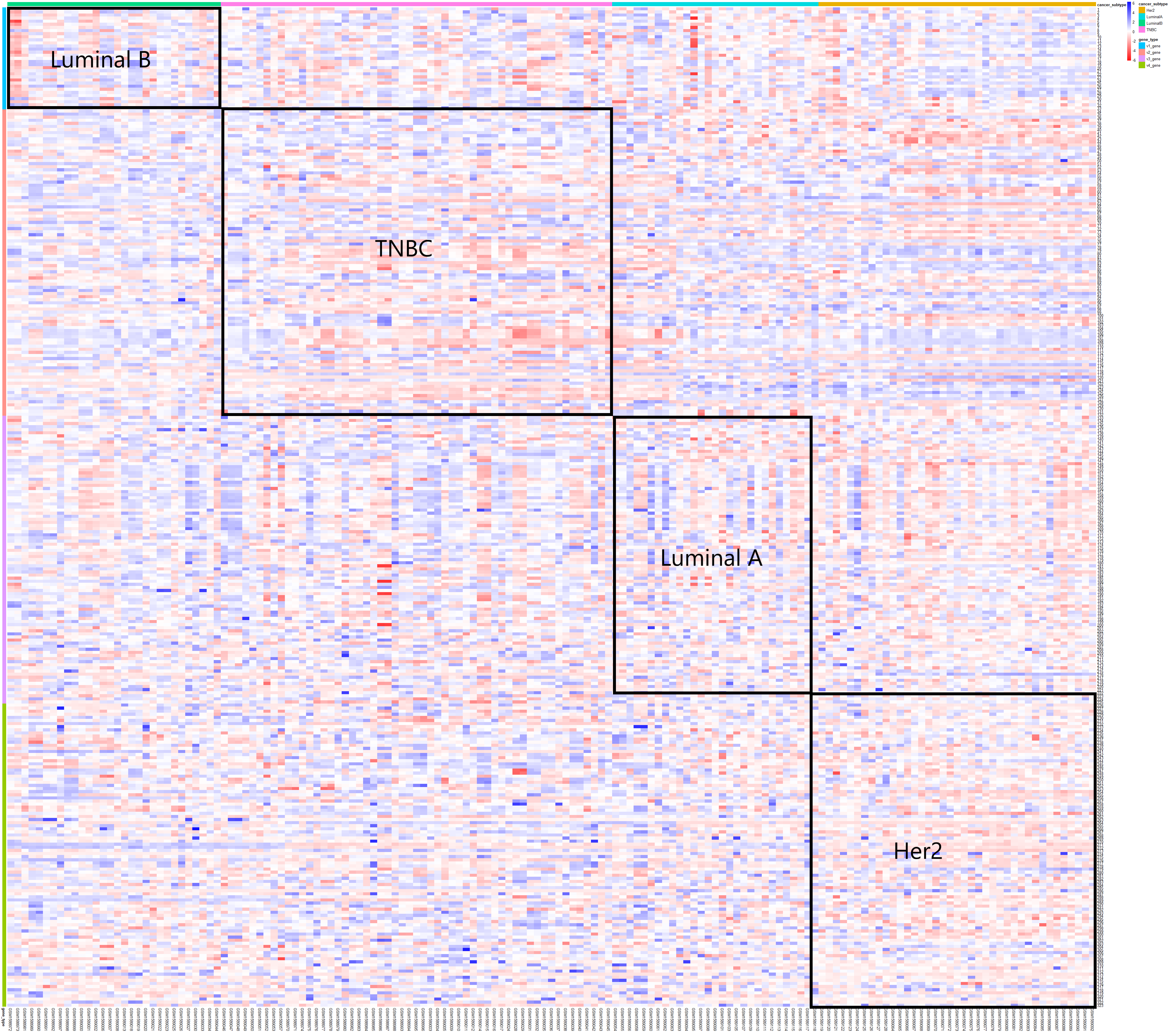


**Supplementary Figure S5** Heatmap of BCII by SPCA.


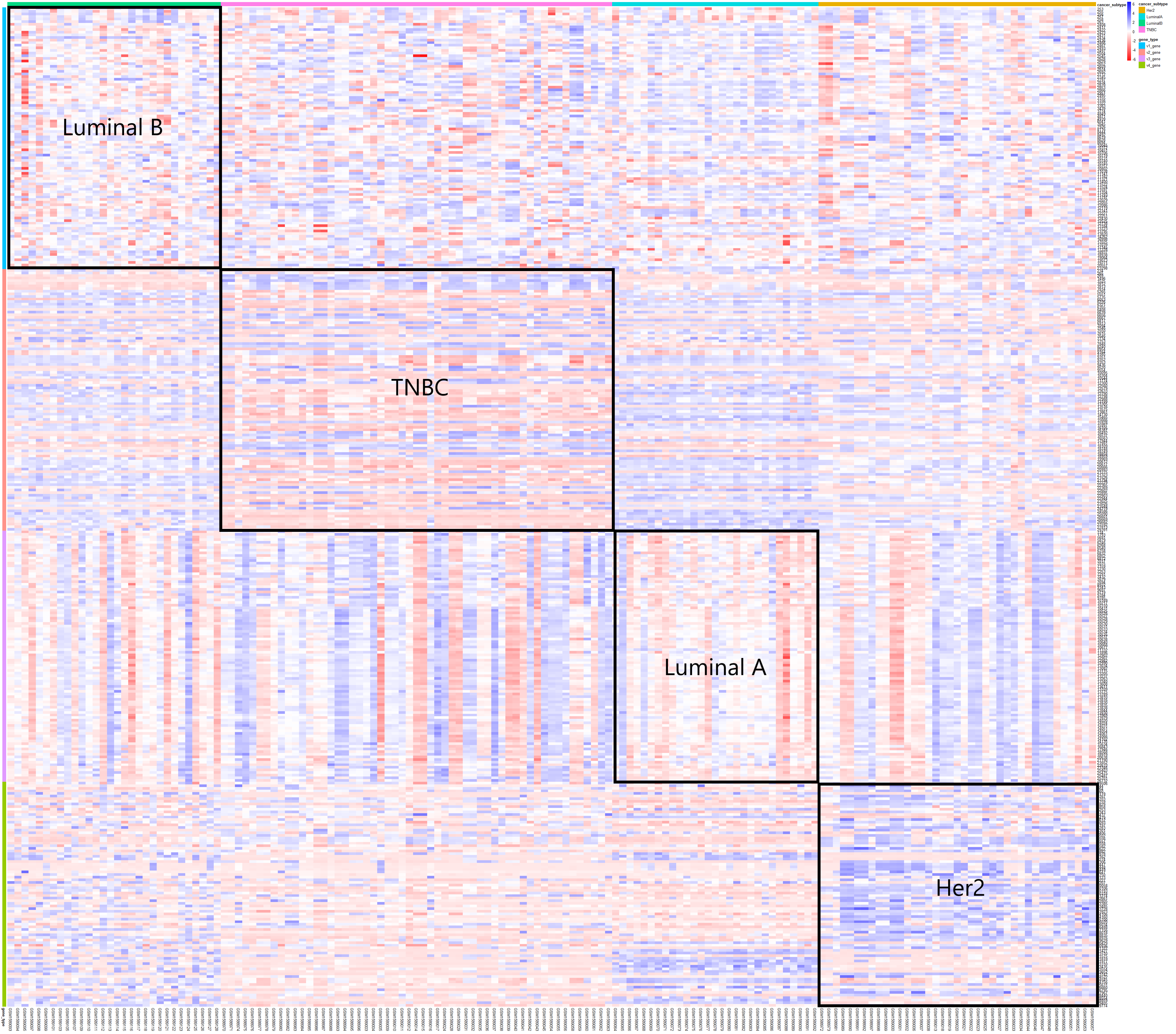


**Supplementary Figure S6** Heatmap of GC by ESPCA.


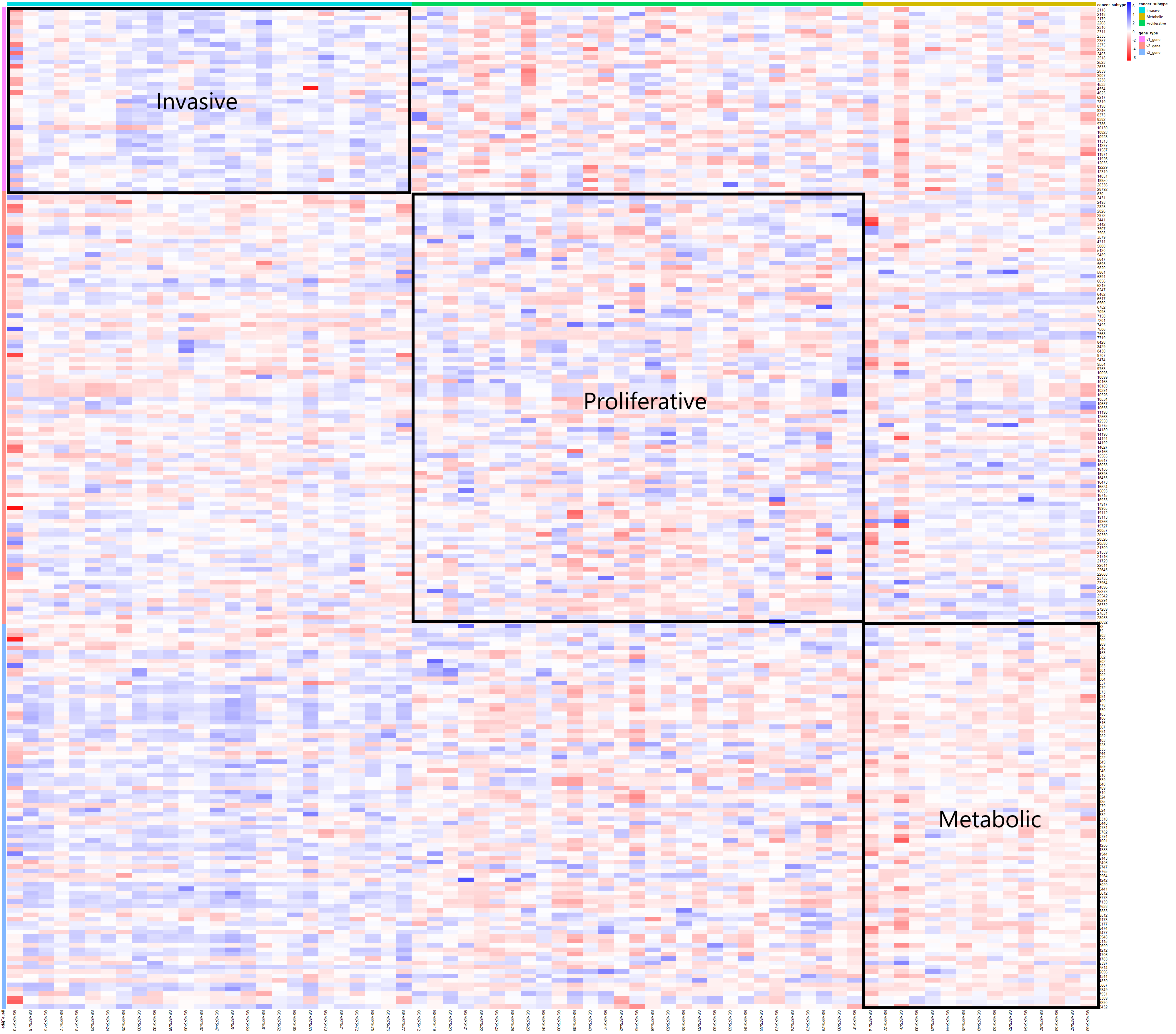


**Supplementary Figure S7** Heatmap of GC by SPCA.


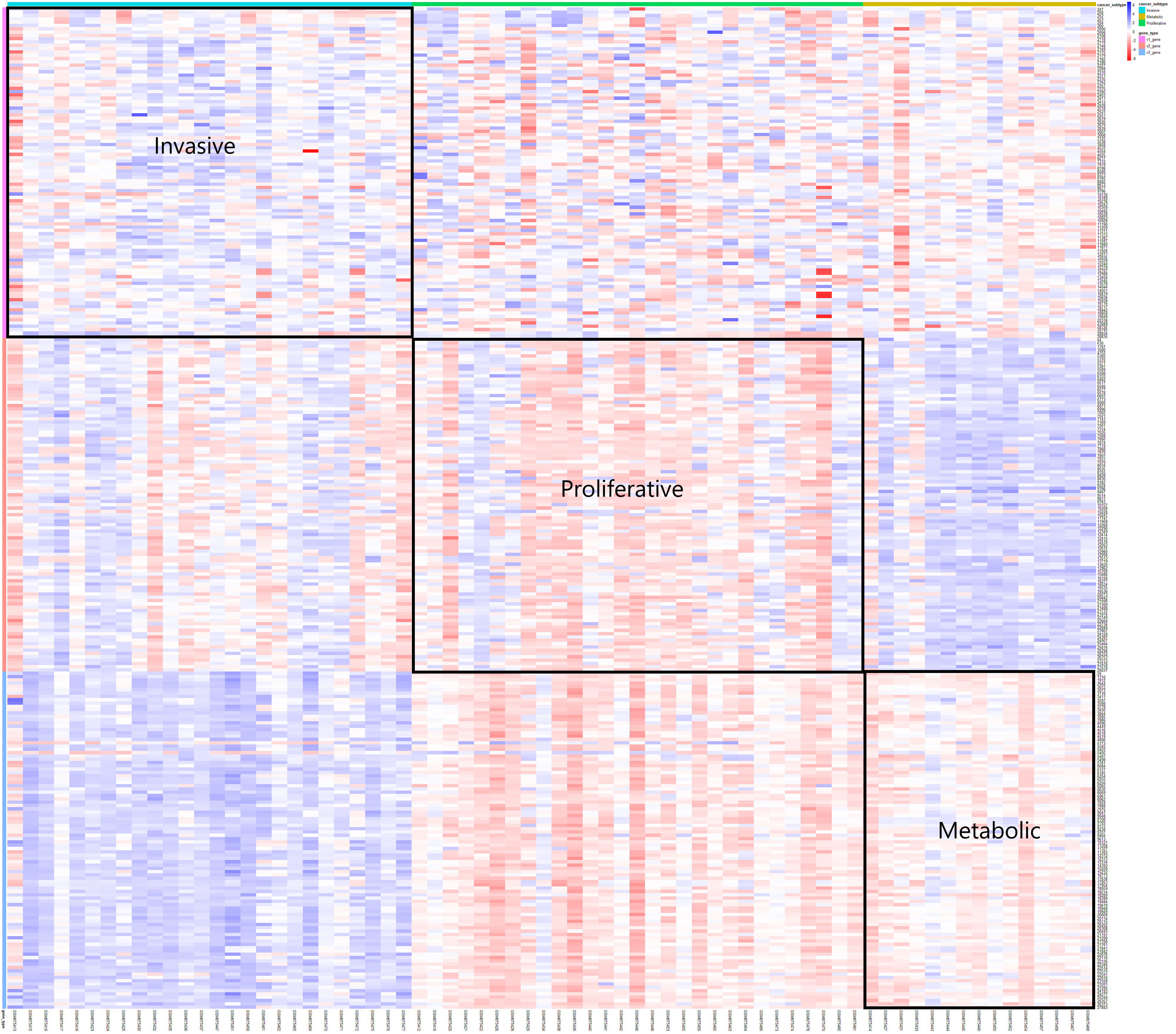


**Supplementary Figure S8** z-score of BCI basal subtype. The figure shows the genes with the highest z-score which related to breast cancer in gene enrichment analysis.


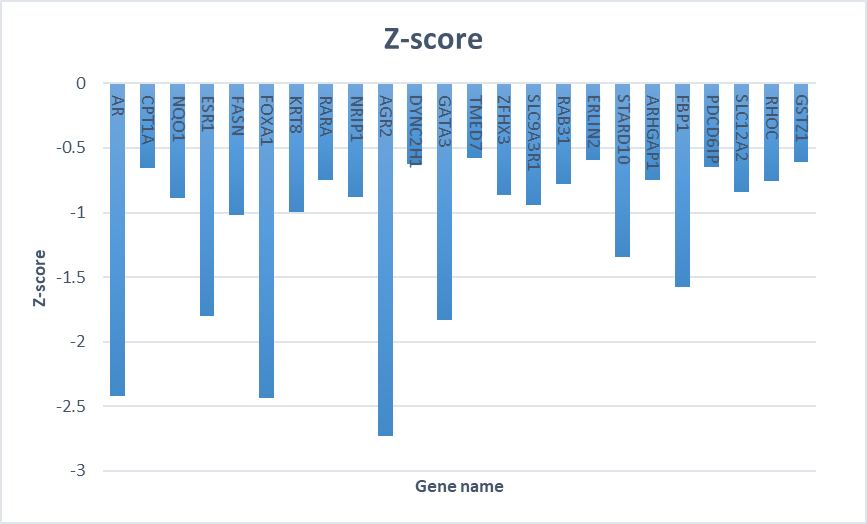


**Supplementary Figure S9** the relationship between diseases and gene probes selected by DM-ESPCA model in BCII Luminal B subtype according to gene enrichment analysis.


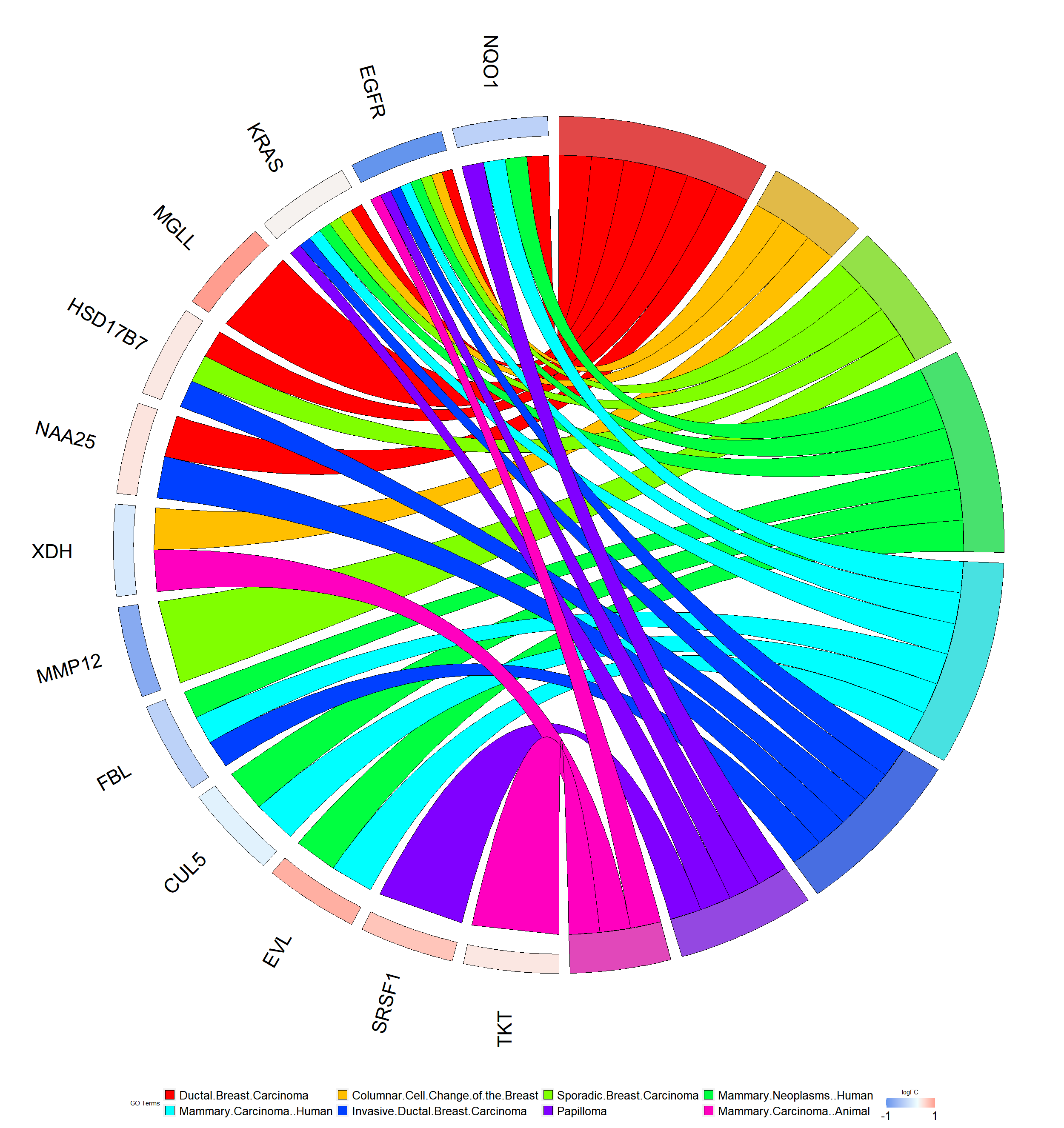


**Supplementary Figure S10** p-values of Luminal A and all subtypes of BCI.


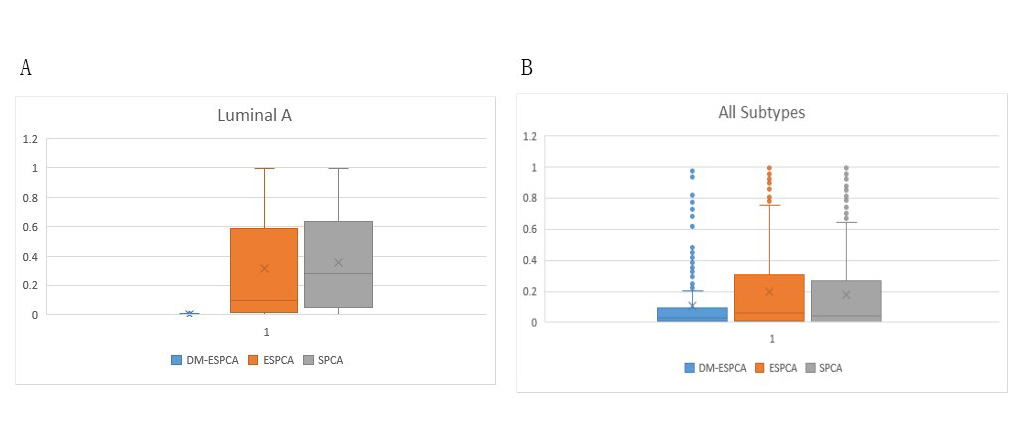


**
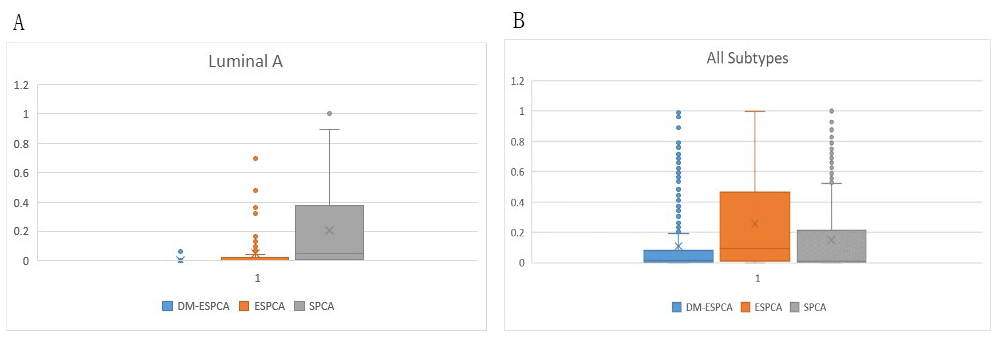
Supplementary Figure S11** p-values of Luminal A and all subtypes of BCII.

**Supplementary Figure S12** the results of KNN in three sparse PCA models and all gene of BCII.


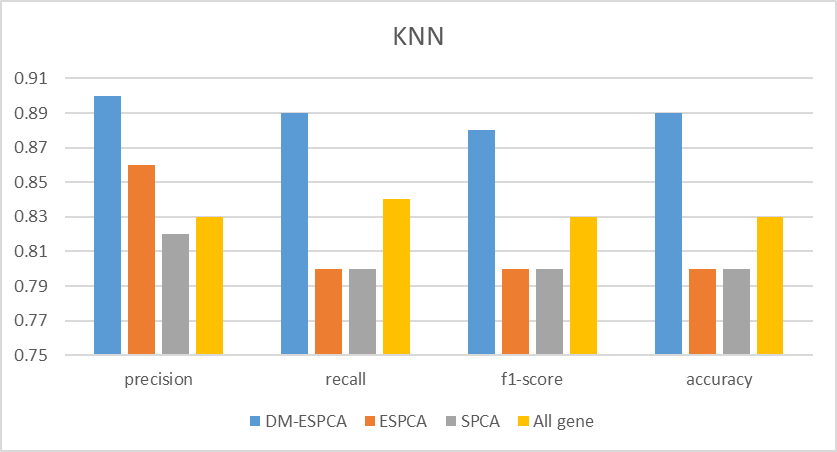


**
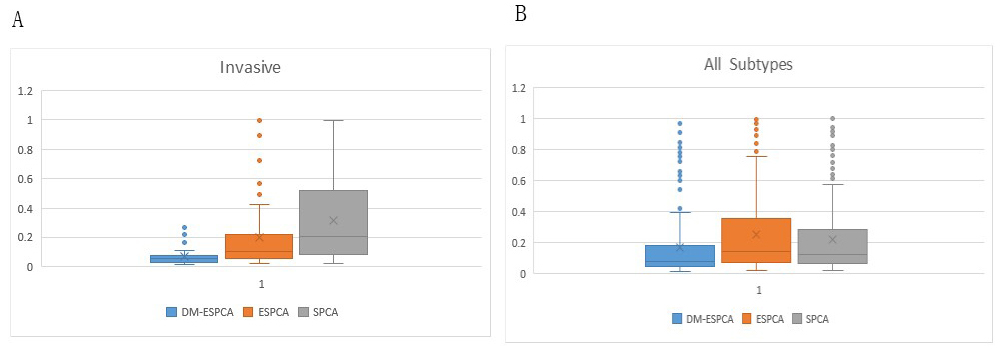
Supplementary Figure S13** P-values of Invasive and all subtypes of GC.

**Supplementary Figure S14** the results of KNN in three sparse PCA models and all gene of GC.


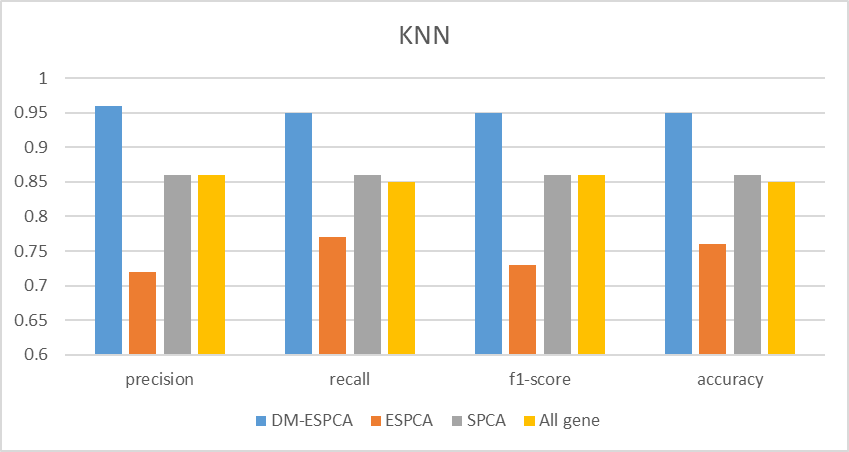


**Reference**

[1] M.-W. Huang, C.-W. Chen, W.-C. Lin, S.-W. Ke, and C.-F. Tsai, SVM and SVM ensembles in breast cancer prediction. PloS one 12 (2017) e0161501.

[2] Y. Rejani, and S.T. Selvi, Early detection of breast cancer using SVM classifier technique. arXiv preprint arXiv:0912.2314 (2009).

[3] C. Nguyen, Y. Wang, and H.N. Nguyen, Random forest classifier combined with feature selection for breast cancer diagnosis and prognostic. (2013).

[4] S.A. Medjahed, T.A. Saadi, and A. Benyettou, Breast cancer diagnosis by using k-nearest neighbor with different distances and classification rules. International Journal of Computer Applications 62 (2013).

[5] K. Odajima, and A.P. Pawlovsky, A detailed description of the use of the kNN method for breast cancer diagnosis, 2014 7th International Conference on Biomedical Engineering and Informatics, IEEE, 2014, pp. 688-692.

**
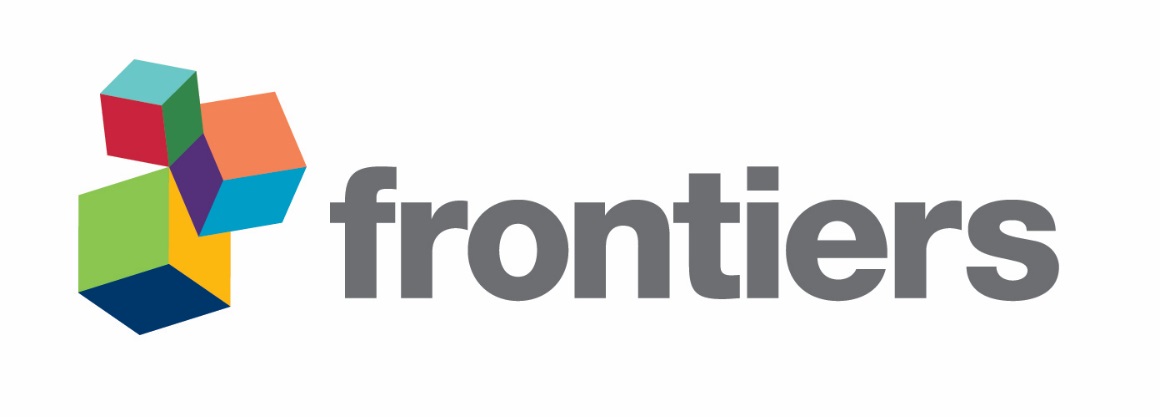
**
